# Medical Students’ Knowledge of Fertility Awareness-Based Methods of Family Planning

**DOI:** 10.3389/fmed.2017.00065

**Published:** 2017-06-01

**Authors:** Peter G. Danis, Sally A. Kurz, Laura M. Covert

**Affiliations:** ^1^Mercy Family Medicine Residency St. Louis, St. Louis, MO, United States; ^2^Mercy Clinic East Community St. Louis, St. Louis, MO, United States

**Keywords:** fertility awareness-based method, natural family planning methods, methods of family planning, medical student education, female reproductive physiology, fertility awareness

## Abstract

**Objective:**

Traditional medical school curricula have not addressed fertility awareness-based methods (FABMs) of family planning. The objective of this study was to assess (1) 3-year medical students’ knowledge of FABMs of family planning, (2) their confidence in utilizing that knowledge in patient care, and (3) to implement focused education on FABMs to improve knowledge and confidence.

**Methods:**

Third-year medical students at one institution in the United States were given a 10-question assessment at the beginning of their OB-GYN rotation. Two lectures about FABMs and their clinical applications were given during the rotation. Students were given the same questions at the end of the rotation. Each questionnaire consisted of eight questions to assess a student’s knowledge of FABMs and two questions to assess the student’s confidence in sharing and utilizing that information in a clinical setting. McNemar’s test was used to analyze the data.

**Results:**

Two hundred seventy-seven students completed a pretest questionnaire and 196 students completed the posttest questionnaire. Medical knowledge improved from an initial test score of 38.99% to final test score of 53.57% (*p* < 0.05). Confidence in sharing FABM information with patients (0 = very uncomfortable; 5 = very comfortable) improved from 1.51 to 3.00 (*p* < 0.05). Confidence in utilizing FABM to diagnose and treat gynecologic/reproductive problems (0 = not very confident and 5 = very confident) improved from 1.01 to 3.15 (*p* < 0.05).

**Conclusion:**

Medical schools may not include FABMs in OB-GYN curriculum; however, to patients, these methods remain a sought after and valid form of family planning. This study shows that brief, focused education can increase medical students’ knowledge of and confidence with FABMs of family planning.

## Introduction

Fertility awareness-based methods (FABMs) of family planning allow women to monitor biomarkers (i.e., cervical mucus) and identify days of fertility, which can be used to avoid or achieve pregnancy. Physicians with knowledge of these methods can use a woman’s chart to assist in the diagnosis and treatment of gynecologic problems including PMS, abnormal bleeding, and infertility (1–3).

A common misconception is that these methods are not effective. The frequently reported 24% failure rate for FABMs is taken from the 1995 and 2002 National Survey of Family Growth. These retrospective surveys asked women to recall which family planning method they were using at the time of conception. Data for all FABMs were then pooled, including the older and outdated rhythm method, which is not really an FABM as it requires no awareness of fertility at all. In the National Survey of Family Growth, 86% of FABM users reported using this outdated method (4). Combining data from lower quality retrospective surveys for all FABMs, including outdated methods, has led to a misunderstanding of effectiveness of the newer, modern FABMs. Effectiveness is also often underestimated by counting any pregnancy as “failure” of the method, even if a couple chose to have intercourse on a fertile day. This is not a failure of the method, but rather confirmation that these methods can help a couple to accurately identify the fertile window. Studies of modern FABMs, including the Creighton Model Fertility Care System, the Marquette Model, and a symptothermal method, show that the typical unintended pregnancy rates can be comparable to other commonly used contraceptives (5–7).

Many women will seek information about family planning from their physicians, including information about FABMs (8–10). In one study, one in five women in the United States expressed interest in using FABMs when informed about such options (5). In another study, 23% of family medicine residents said patients had inquired about FABMs (9). In order to assist patients in making informed decisions, family physicians need to have accurate information and feel confident in sharing that information with patients. Many physicians do not have this information or confidence, due in part to lack of structured education during medical school (9, 11, 12). One study noted that 27% of family medicine residents reported no time spent and 56% reported less than 1 h spent on FABM education during school (9). Many medical schools in the US utilize the Association of Professors of Gynecology and Obstetrics Medical Student Educational Objectives to teach 3-year students about methods of contraception. There is no mention of FABMs in those objectives or in the associated case discussions. While there may be similar gaps in knowledge regarding other methods of contraception, no other studies exist at this time, which identify a need for improved education in that area.

Women trust family physicians to provide accurate information about family planning so they can choose the best method to meet their needs and values. Family physicians play an integral role in the education and mentoring of students, and this role should include the area of family planning.

Traditional medical school curricula do not include information about FABMs. Therefore, a study was designed to assess 3-year medical students’: (1) knowledge of FABMs, (2) confidence in utilizing that knowledge in patient care, and (3) knowledge and confidence in FABM use after implementation of a focused educational intervention.

## Materials and Methods

**Figure 1:**
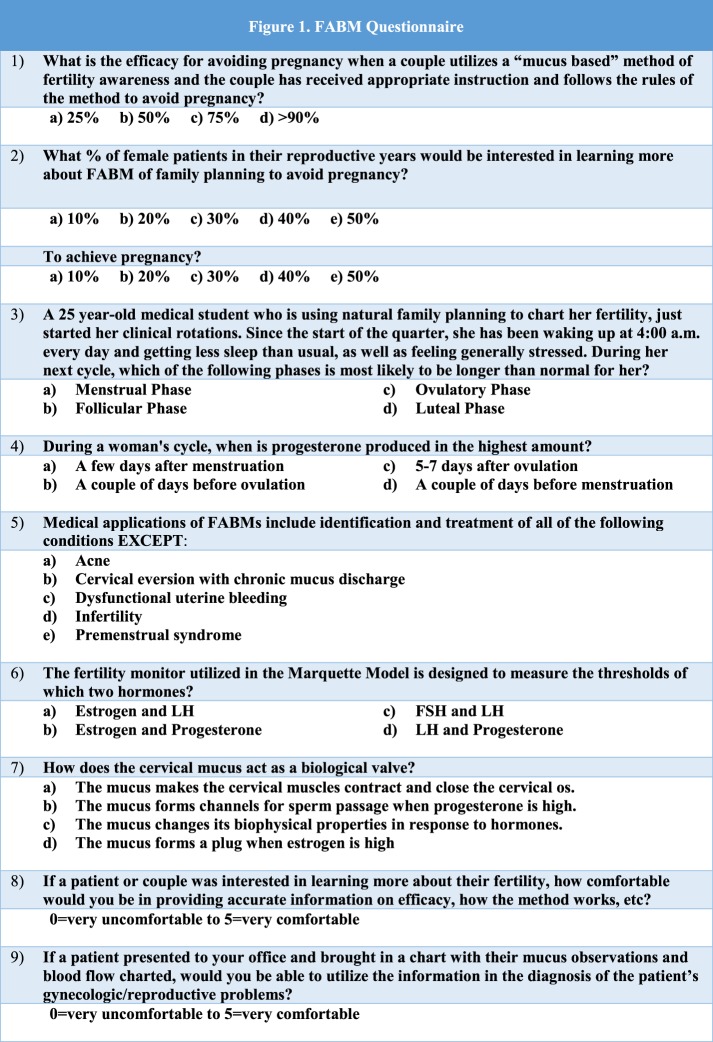
**Fertility awareness-based method (FABM) questionnaire**.

Over a 2-year period (2013–2015), medical students were asked to complete a pretest and a posttest during their 3-year OB-GYN rotation. The pretest and posttest had the same 10 questions: eight assessing medical knowledge and two assessing student confidence in applying that knowledge (Figure 1). The test was created by two board-certified family physicians who were also experts in FABMs and therefore had face validity. Test validity was difficult to obtain beyond face validity at this stage due to the novelty of our study. The students were provided two lectures: (1) Fertility Awareness and Helping Couples Achieve Results They Want and (2) Medical and Surgical Applications of FABMs. The first lecture included review of normal reproductive physiology, the science behind modern FABMs, practical applications of FABMs in family planning, and case discussions regarding use of patients’ FABM charting to solve gynecologic and reproductive problems. The second lecture focused on the use of FABM charting in the diagnosis and treatment of gynecological conditions such as endometriosis and infertility. A family physician and an obstetrician gynecologist, both of whom were trained in the use and application of FABMs, provided the teaching. After the first lecture, students were provided additional resources on the department website (lecture presentations, physician and patient education handouts). These resources contained information needed to answer the test questions correctly, but students were not directly provided the answers. The posttest was administered at the end of the rotation to determine whether students’ knowledge and confidence changed. McNemar’s test was used to analyze the data. This study qualified to be IRB waived.

## Results

**Figure 2:**
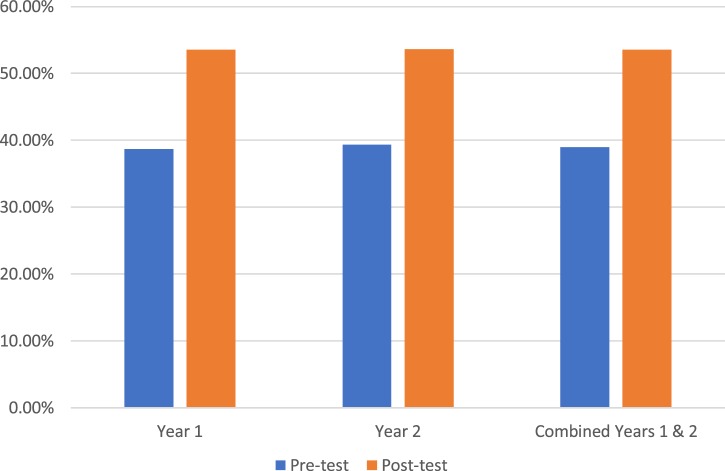
**Average test scores—medical knowledge questions 1–7 (percent correct)**.

**Figure 3:**
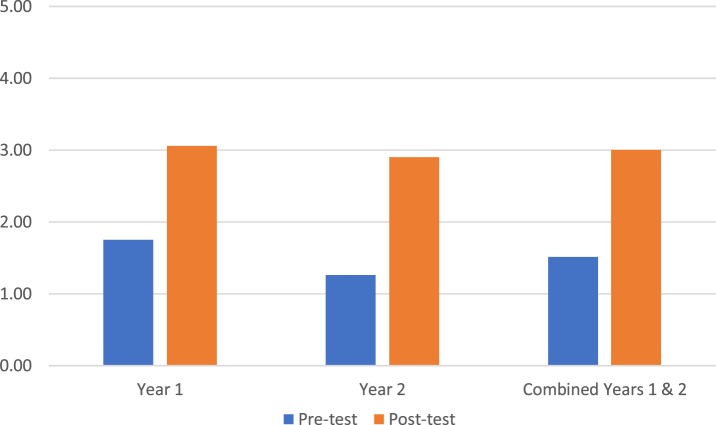
**Question 8: average confidence scores in sharing fertility awareness-based method information (0 = very uncomfortable; 5 = very comfortable)**.

**Figure 4:**
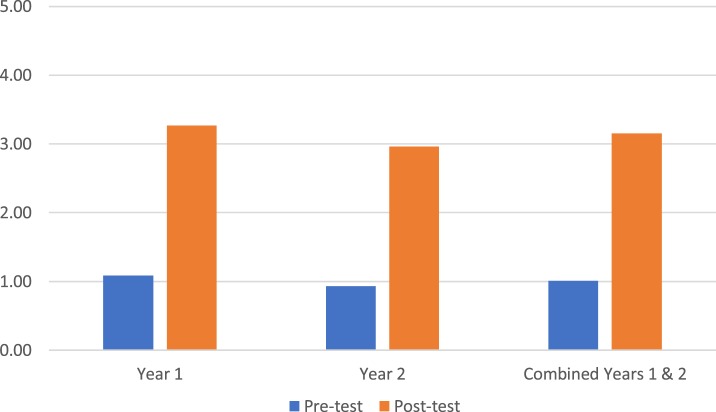
**Question 9: average student confidence ratings in utilizing fertility awareness-based method to diagnose and to treat (0 = not very confident and 5 = very confident)**.

Students’ test scores on medical knowledge of FABMs (questions 1–7) averaged 38.99% on the pretest (*n* = 277) and 53.57% on the posttest (*n* = 196; *p* < 0.05; Figure 2). Students’ test scores on sharing FABM information with a patient/couple (question 8) showed an average student confidence pretest rating of 1.51 versus a posttest rating of 3.00 (*p* < 0.05; Figure 3). Student test scores on utilizing FABMs to diagnose and treat gynecologic problems (question 9) showed an average student confidence pretest rating of 1.01 versus a posttest rating of 3.15 (*p* < 0.05; Figure 4).

## Discussion

This study began with an assessment of medical students’ knowledge of FABMs and their self-reported confidence. Through the application of a focused set of resources, we demonstrated a positive effect on student knowledge and confidence.

Our study had several limitations:

- Because of call schedules and L&D responsibilities, students may not have taken both tests. Some could have missed the pretest and lecture and taken the test for the first time on the last day of the rotation (the posttest). Some students who took the pretest and heard the first lecture may have missed the opportunity to have completed the posttest. If we had assigned every student a unique identifier and allowed for electronic completion, we may have seen a more positive effect by our intervention.
- Curriculum could have been focused to meet specific learning objectives (currently there are no learning objectives defined for this area of the OB-GYN curriculum) so that questions could be more accurately designed to assess knowledge and attitudes.
- These results may not be predictive of long-term retained knowledge and confidence or changes in clinical behavior.

Although this study focused on education of 3-year students, FABMs could also be incorporated into the hormonal and reproductive physiology that is taught in the preclinical years. Through an understanding of fertility awareness charting and how it applies to normal physiology, students could then apply these principles to assist patients that they will see in the clinical years. This would include offering accurate information on FABMs to patients who were interested in discussing family planning options. FABMs can be taught to patients in a variety of ways, including classes, one-on-one teaching by certified instructors, and online learning. This study was not designed to make medical students instructors of FABMs, but rather to provide accurate information to medical students that would enable them to provide resources to interested patients as well as assisting them in solving medical problems. Medical students can be taught to use the patient’s FABM chart to diagnose and treat gynecologic and reproductive problems. For example, one set of protocols (called NaProTechnology) has been developed based on the Creighton Model Fertility Care System; this can be used by physicians to assist their patients in the diagnosis and treatment of a variety of medical problems, including abnormal bleeding, PMS, infertility, and recurrent miscarriage (3, 13).

The following example can help to explain the importance of the concept of using a patient’s FABM chart to solve a medical problem. In women with symptoms of PMS or abnormal bleeding, certain patterns of bleeding observed on a woman’s FABM chart can be associated with hormonal abnormalities (13). Accurate hormonal assessment is based on a properly timed evaluation. A woman tracking her cycles with an FABM can confidently identify her “peak day” of mucus (last day of clear, stretchy, or lubricative mucus) which is closely associated with the day of her ovulation (13). If the physician desires a mid-luteal hormone test, this can be accurately done based on her identification of day of ovulation, or “peak” day, which approximates the beginning of the luteal phase. This is a far more accurate assessment than what is done by simply obtaining a blood test that is presumed to be mid-luteal (typically “day 21”) based on a patient’s menstrual bleeding. In addition to the diagnostic advantages, treatment can then be instituted in a properly timed fashion to correct the underlying disorder working with the patient in a cooperative manner. The patient’s continued observations and charting can then allow her and the physician to monitor the effect of the treatment.

Although this study is one of the first of its kind, we believe that Departments of Family Medicine and Obstetrics and Gynecology can collaborate to develop objectives and methods (formal teaching, web-based learning, etc.) for teaching the science and clinical applications of FABMs. ACOG published a patient education pamphlet on FABMs in 2015^1^ and yet there is still very little education on FABMs in medical school curricula. Organizations like Fertility Appreciation Collaborative to Teach the Science^2^ and the International Institute for Restorative Reproductive Medicine^3^ are working to improve the dissemination of this knowledge. Case discussions involving methods of family planning, including FABMs, could also be used in cultural competency curricula as a way of exploring the importance of culture, values, and religion in such decisions. The inclusion of FABMs in medical student education can reinforce basic science and clinical medicine and better prepare future physicians to meet the family planning needs of patients.

## Author Contributions

PD: investigator, coauthor, and lecturer. SK: coauthor. LC: investigator and coauthor.

## Conflict of Interest Statement

The authors declare that the research was conducted in the absence of any commercial or financial relationships that could be construed as a potential conflict of interest.

## Footnotes

^1^www.acog.org/Patients/FAQs/Fertility-Awareness-Based-Methods-of-Family-Planning.

^2^www.factsaboutfertility.org.

^3^http://restorative-reproductive-medicine.com.
